# Bilateral robotic sympathectomy for recurrent ventricular tachycardia in a patient with complex cardiovascular history

**DOI:** 10.1093/jscr/rjag788

**Published:** 2026-09-02

**Authors:** John J Tumminello, Alexander Pohlman, Zaid M Abdelsattar, Julia M Coughlin

**Affiliations:** Stritch School of Medicine, Loyola University Chicago, 2160 S 1st Ave, Maywood, IL 60153, United States; Department of Cardiovascular and Thoracic Surgery, Loyola University Medical Center, 2160 S 1st Ave, Maywood, IL 60153, United States; Department of Surgery, University of Illinois Chicago, 840 S. Wood St Chicago, IL 60612, United States; Stritch School of Medicine, Loyola University Chicago, 2160 S 1st Ave, Maywood, IL 60153, United States; Department of Cardiovascular and Thoracic Surgery, Loyola University Medical Center, 2160 S 1st Ave, Maywood, IL 60153, United States; Department of Surgery, Edward Hines Jr. Veterans Affairs Hospital, 5000 5th Ave, Hines, IL 60141, United States; Stritch School of Medicine, Loyola University Chicago, 2160 S 1st Ave, Maywood, IL 60153, United States; Department of Cardiovascular and Thoracic Surgery, Loyola University Medical Center, 2160 S 1st Ave, Maywood, IL 60153, United States

**Keywords:** refractory ventricular tachycardia, sympathectomy, stellate ganglion block, robotic surgery

## Abstract

A 76-year-old female with complex cardiovascular history requiring multiple open and transcatheter interventions presented with recurrent ventricular tachycardia (VT) and multiple implantable cardioverter-defibrillator (ICD) shocks. Her VT persisted despite maximal medical therapy. She underwent two left stellate ganglion blocks, which provided temporary VT suppression. She was found to have significant cardiomegaly and a large chronic descending aortic dissection on pre-operative workup, which did not require acute intervention. Given her persistent symptoms, she underwent bilateral robotic sympathectomy. She tolerated the procedure without complication and remained without ICD shocks at her 6-month clinic visit.

## Introduction

Ventricular tachycardia (VT) refractory to antiarrhythmic drugs, stellate ganglion blocks (SGB), and catheter ablation therapies presents a significant therapeutic challenge, particularly in high-risk patients with complex structural heart disease and prior open-heart operations [1–5]. Cardiac sympathetic denervation (CSD) by way of bilateral sympathectomy has been shown to reduce arrhythmia burden in such cases by interrupting sympathetic input to the heart [2, 3]. Robotic-assisted bilateral sympathectomy (RA-BS) offers enhanced visualization and facilitates expeditious dissection in hostile pleural spaces compared to open or video-assisted thoracoscopic approaches [5].

To our knowledge, reports of RA-BS performed as a single-staged operation in such high-risk patients are rare. This case demonstrates that RA-BS is a safe and effective approach to achieving definitive VT suppression in appropriately selected high-risk patients.

## Case report

A 76-year-old female with atrial fibrillation, severe mitral regurgitation (MR) status post mechanical mitral valve replacement (MVR) and biventricular implantable cardioverter-defibrillator (ICD), complicated by infective endocarditis requiring redo sternotomy with MVR explant and bioprosthetic replacement, ICD and lead extraction, epicardial lead placement, further complicated by severe MR status post MitraClip presented with recurrent VT and multiple ICD shocks. Her VT persisted despite maximal intravenous medical therapy. She underwent a left SGB. Three weeks later, she presented with recurrent VT and ICD shocks. Echocardiography showed an acute reduction in her ejection fraction (EF) from 59% to 22%. She was not a candidate for ablative therapy. To allow for recovery of her EF and cardiac optimization, she underwent a repeat left SGB, with plan for a bilateral sympathectomy during the same hospitalization. She had no further episodes of VT and her EF improved to 45%. A pre-operative computed tomography (CT) chest demonstrated significant cardiomegaly and a large chronic descending aortic dissection, which did not require intervention. Given the likelihood of significant adhesions and anatomic complexity of the left pleural space, we elected to perform a right-sided sympathectomy first.

**Figure 1:**
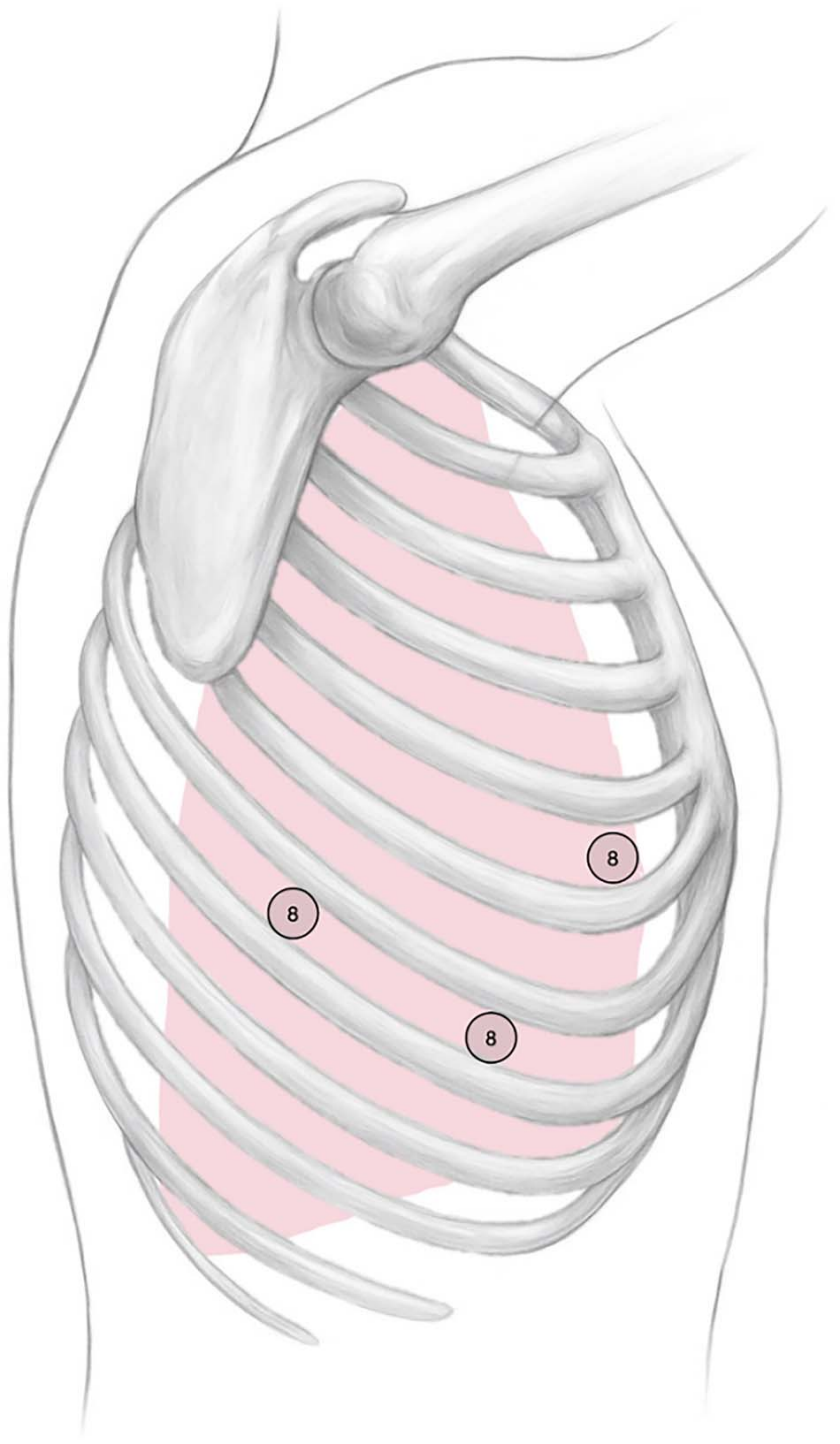
Right-sided robotic port placement.

The patient was placed supine on the operating table. The ICD was deactivated, and external defibrillator pads were placed. General anesthesia was induced with a double-lumen endotracheal tube. The patient was placed in the left lateral decubitus position. Three 8 mm robotic ports were placed: two in the sixth intercostal space anterior to the scapular tip and in the anterior axillary line; and one in the fourth intercostal space in the anterior axillary line (Fig. 1). The pleural space was carefully insufflation to a pressure of 8 mm Hg, with close observation of the patient’s hemodynamics.

**Figure 2:**
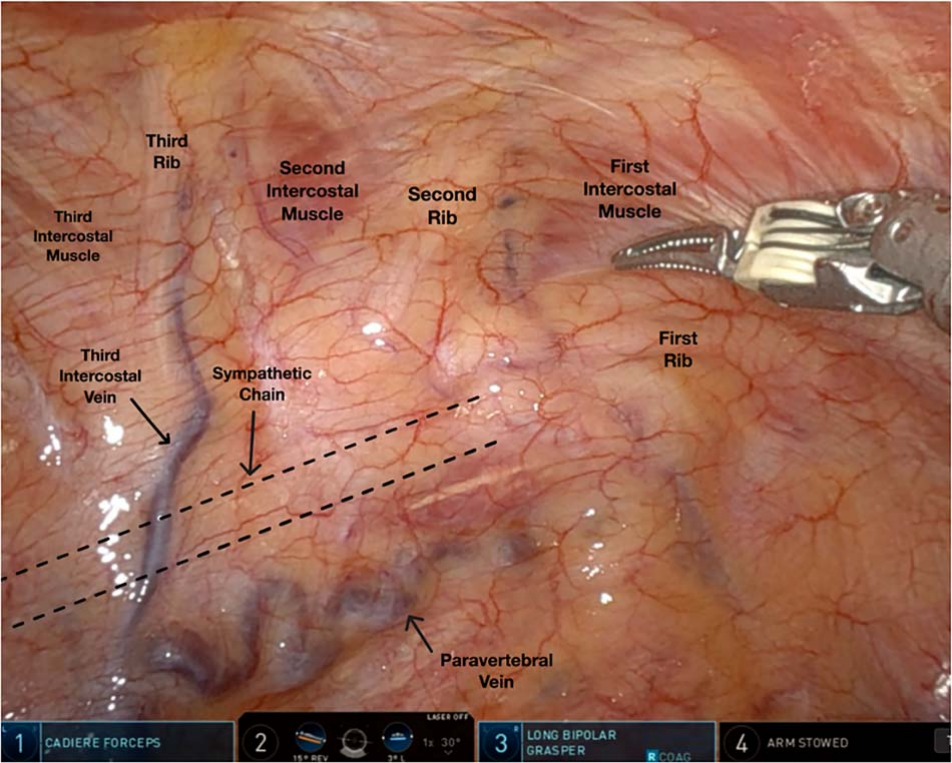
Initial exposure of the right-sided sympathetic chain.

**Figure 3:**
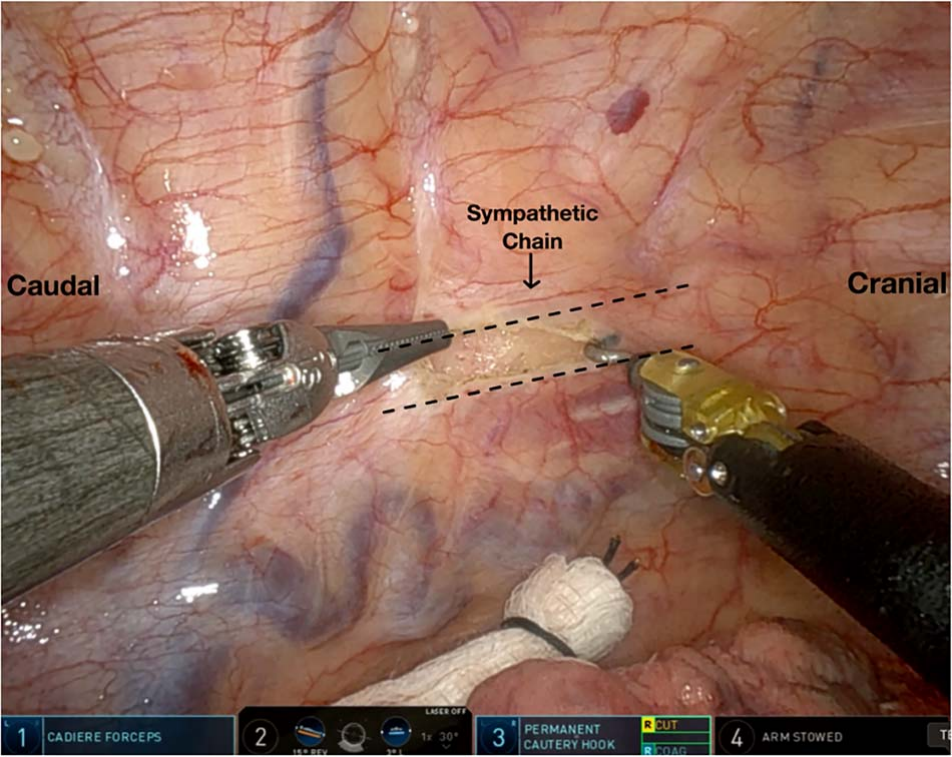
Incising the parietal pleura overlying the right sympathetic chain.

**Figure 4:**
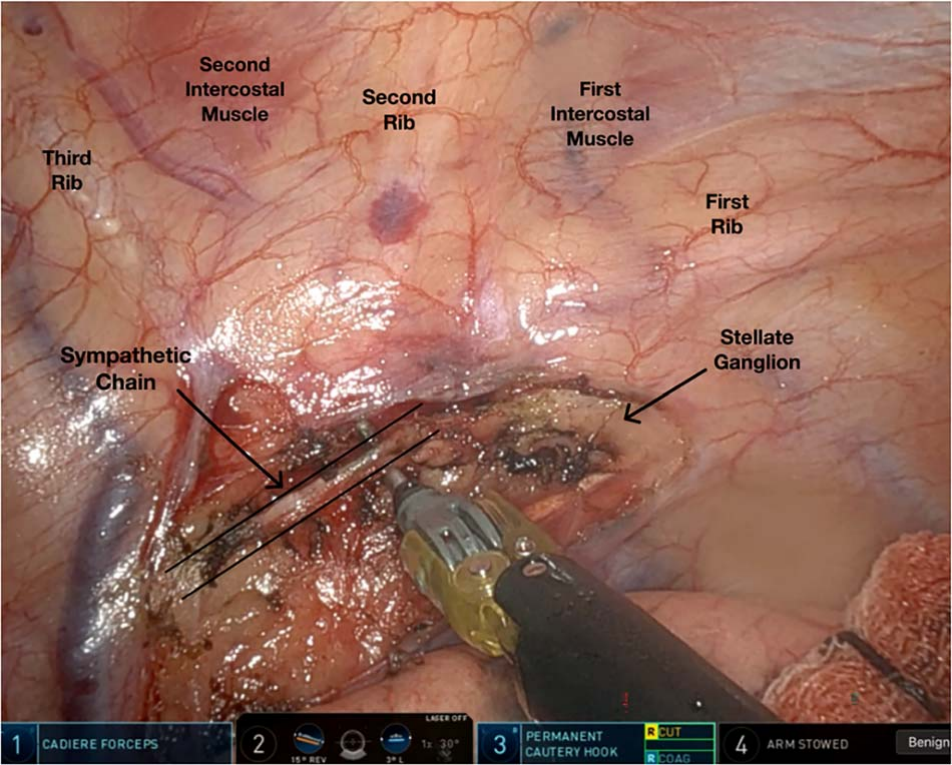
Exposure of the right sympathetic chain and stellate ganglion.

**Figure 5:**
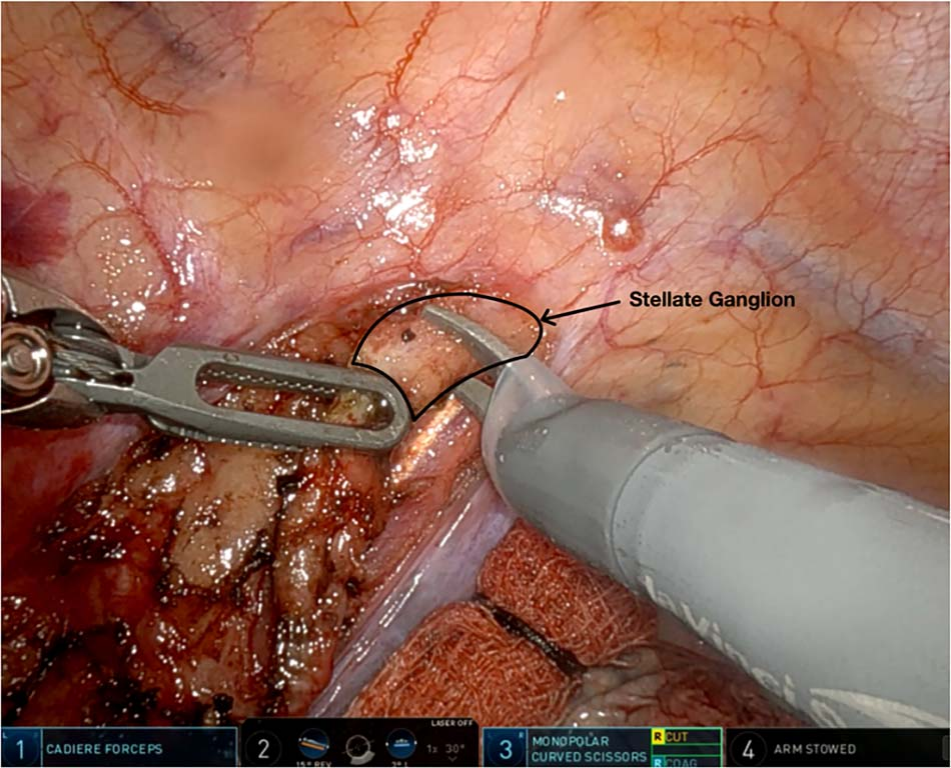
Division of the lower one-third of the stellate ganglion.

**Figure 6:**
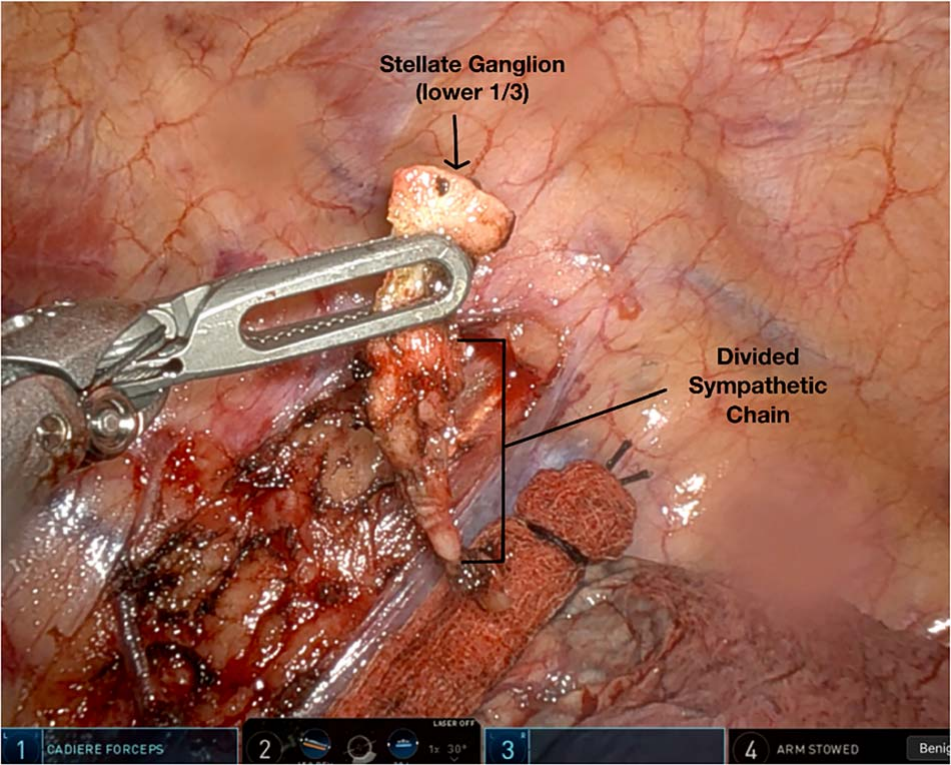
Divided right sympathetic chain (top of R1 - top of R4) and lower one-third of the stellate ganglion.

**Figure 7:**
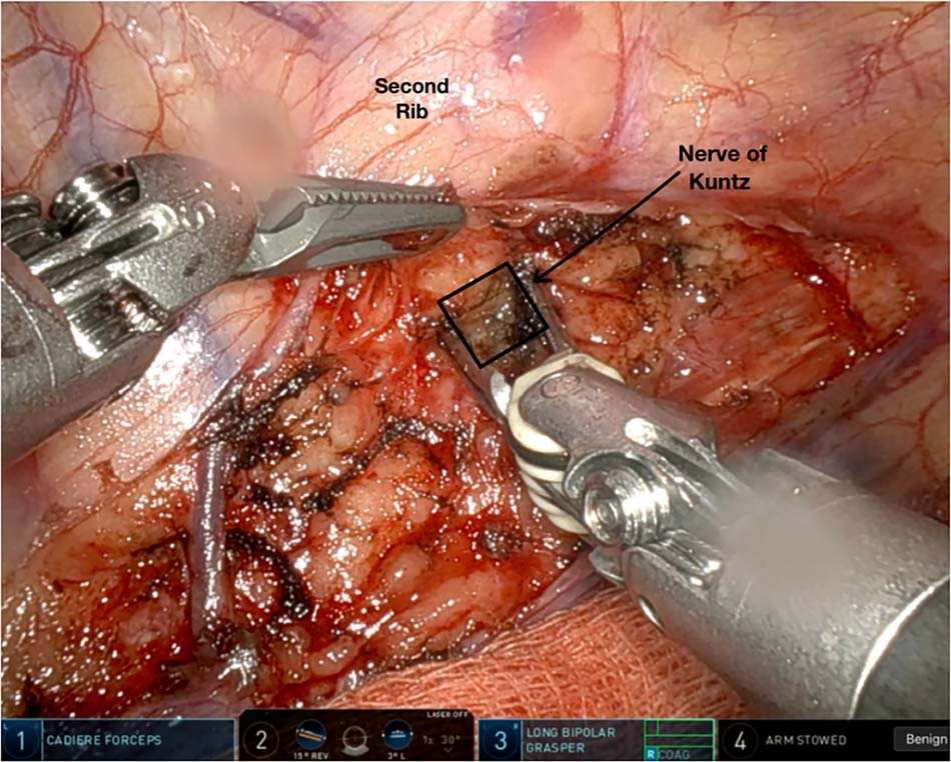
Division of the nerve of Kuntz at the base of the right second rib.

The lung was retracted anteriorly, and the sympathetic chain was identified lateral to the thoracic vertebrae (Fig. 2). The overlying parietal pleura was divided using hook cautery (Fig. 3). The sympathetic chain was divided sharply at the top of the fourth rib. It was circumferentially dissected superiorly to the lower 1/3 of the stellate ganglion, where it was divided sharply (Figs 4–6). The use of cautery was avoided near the stellate ganglion to avoid Horner’s syndrome. Bipolar cautery was used to divide the nerves of Kuntz, crossing the second and third ribs 2 cm lateral from the transverse process (Fig. 7). Hemostasis was achieved. The lung was re-expanded over a 24Fr Blake drain. The incisions were closed in the standard fashion.

**Figure 8:**
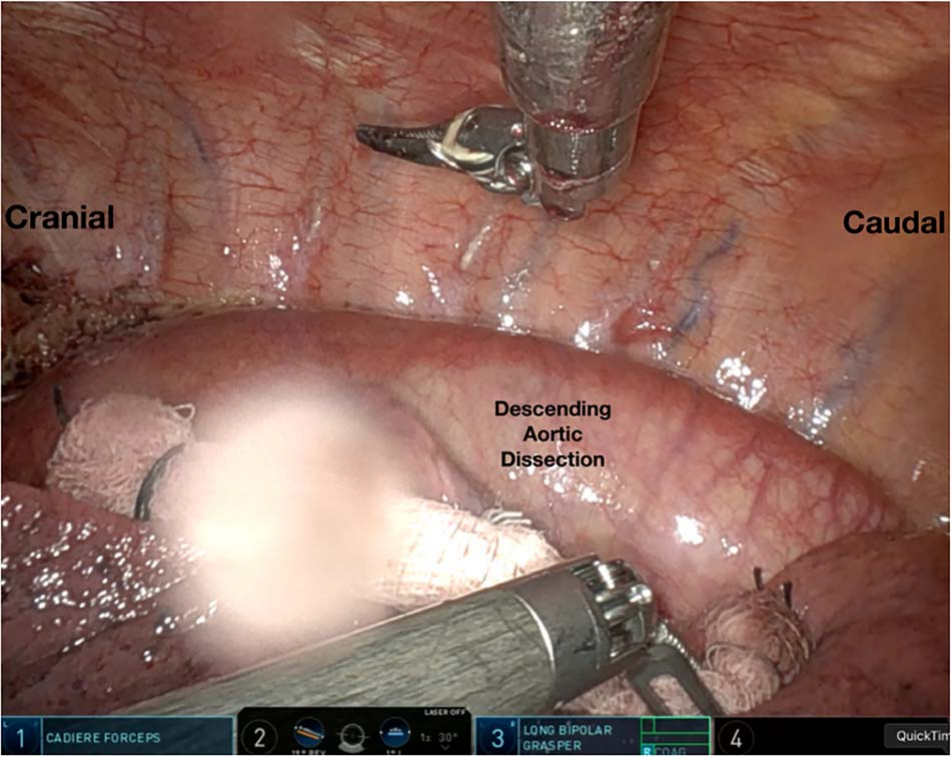
Chronic descending aortic dissection.

**Figure 9:**
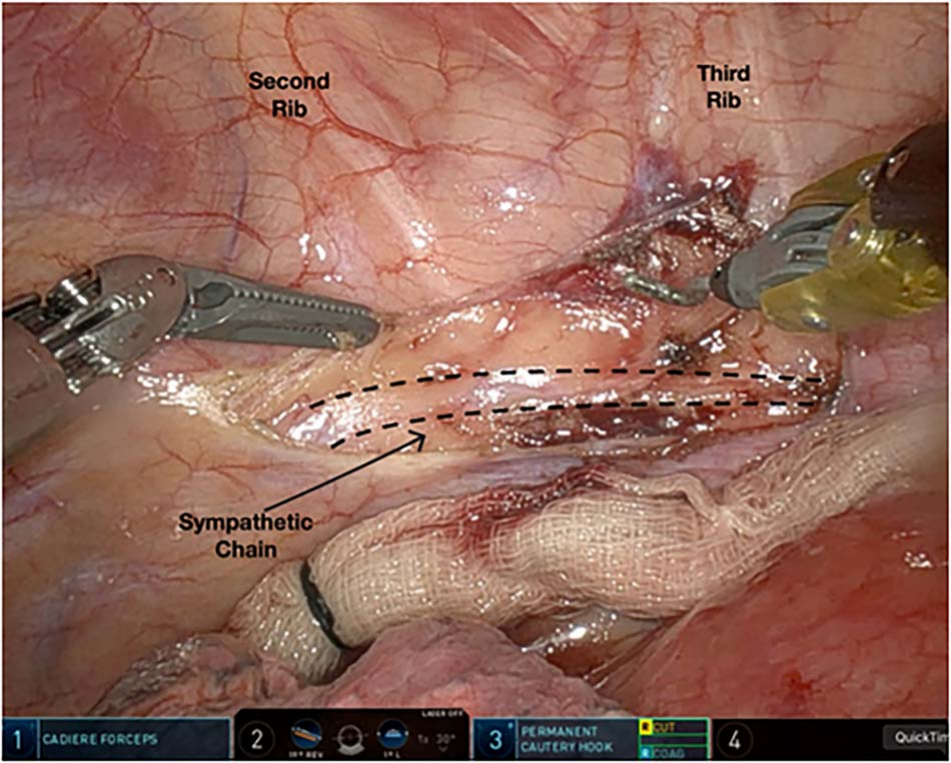
Incising the parietal pleura over the left sympathetic chain.

**Figure 10:**
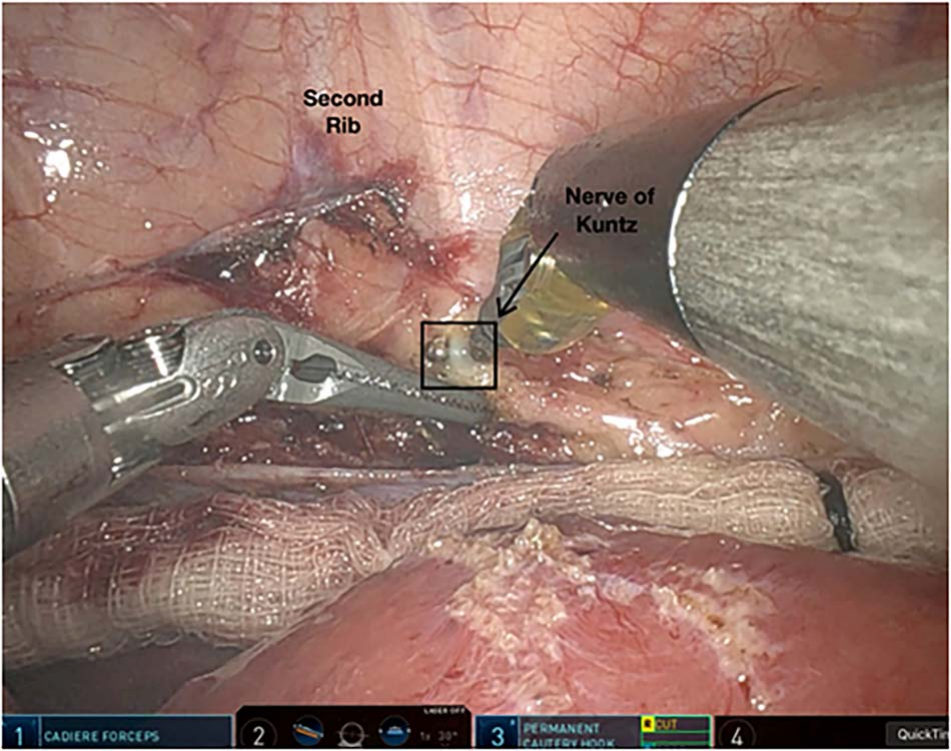
Division of the nerve of Kuntz at the base of the left second rib.

The patient was then placed in the right lateral decubitus position, and ports were placed in a mirror image configuration. Diffuse adhesions throughout the left pleural space were lysed. The descending aorta was significantly enlarged, consistent with the known aortic dissection (Fig. 8). A left sympathectomy was carefully performed in the same fashion as described previously (Figs 9 and 10).

The operative time for each side was 18 and 26 minutes, respectively. The patient tolerated the procedure well without complication. Postoperatively, the patient was closely monitored in the cardiac critical care unit for recurrent VT, postoperative bradycardia, and autonomic instability. The right and left Blake drains were removed on post-operative day 1 and 2, respectively. Due to significant deconditioning from her prolonged pre-operative hospital admission, she was discharged to an inpatient rehabilitation facility on post-operative day 8. She remained free of ICD shocks at her 6-month post-operative visit.

## Discussion

Successful management of refractory VT in high-risk patients with complex cardiothoracic surgical history poses a clinical challenge. Given the significant comorbidities of patients who are prone to developing refractory VT, clinicians often rely on conservative measures and deem surgical intervention “too high-risk.” However, conservative measures often result in variable rates of suppression. SGB has been shown to achieve *short-term* VT suppression in ~60%–70% of patients with frequent recurrence, and catheter ablation provides variable long-term control (50%–70%) [4, 5]. In comparison, surgical CSD offers more consistent and durable reduction in ICD shocks and arrhythmia burden [4, 5].

While left sympathectomy is thought to be more effective than right due to its predominant influence on cardiac electrophysiology, bilateral sympathectomy may confer greater protection in patients with high recurrence risk. Prior studies have shown that patients who underwent left-only sympathectomy had almost twice the risk of recurrent VT [5]. Thus, our typical operative approach in patients undergoing bilateral CSD is to begin on the left side in the event that the patient does not tolerate general anesthesia for the duration of the bilateral procedure. In this patient, however, her surgical history and pre-operative imaging suggested a hostile left pleural space, prompting a right-sided approach first.

Although this patient population is high-risk, minimally invasive surgical intervention should not be excluded from their multidisciplinary discussion. The superior visualization afforded by the robotic platform provides clear identification and division of the sympathetic chain and nerves of Kuntz, ensuring complete denervation and reduced rates of recurrence [4, 6, 7]. In addition, enhanced dexterity allows for efficient dissection, resulting in reduced time under general anesthesia and single-lung ventilation. Eligible patients from this high-risk population need to be carefully selected by a multidisciplinary team consisting of cardiology, electrophysiology, and cardiothoracic surgery. In addition, the surgical and anesthesia teams should be prepared for intra-operative events, such as sustained cardiac arrhythmias and transient hemodynamic instability [6, 7]. Transient postoperative bradycardia may occur following bilateral sympathectomy due to reduced sympathetic cardiac input and should be anticipated during postoperative monitoring. In most cases, this is self-limited and managed conservatively.

This case demonstrates that RA-BS in patients with complex cardiovascular disease and hostile thoracic anatomy is a safe and effective approach in appropriately selected high-risk patients.
